# The Effect of Magnetic Resonance Imaging Based Radiomics Models in Discriminating stage I–II and III–IVa Nasopharyngeal Carcinoma

**DOI:** 10.3390/diagnostics13020300

**Published:** 2023-01-13

**Authors:** Quanjiang Li, Qiang Yu, Beibei Gong, Youquan Ning, Xinwei Chen, Jinming Gu, Fajin Lv, Juan Peng, Tianyou Luo

**Affiliations:** Department of Radiology, The First Affiliated Hospital of Chongqing Medical University, Chongqing 400016, China

**Keywords:** nasopharyngeal carcinoma, cancer staging, magnetic resonance imaging

## Abstract

Background: Nasopharyngeal carcinoma (NPC) is a common tumor in China. Accurate stages of NPC are crucial for treatment. We therefore aim to develop radiomics models for discriminating early-stage (I–II) and advanced-stage (III–IVa) NPC based on MR images. Methods: 329 NPC patients were enrolled and randomly divided into a training cohort (n = 229) and a validation cohort (n = 100). Features were extracted based on axial contrast-enhanced T1-weighted images (CE-T1WI), T1WI, and T2-weighted images (T2WI). Least absolute shrinkage and selection operator (LASSO) was used to build radiomics signatures. Seven radiomics models were constructed with logistic regression. The AUC value was used to assess classification performance. The DeLong test was used to compare the AUCs of different radiomics models and visual assessment. Results: Models A, B, C, D, E, F, and G were constructed with 13, 9, 7, 9, 10, 7, and 6 features, respectively. All radiomics models showed better classification performance than that of visual assessment. Model A (CE-T1WI + T1WI + T2WI) showed the best classification performance (AUC: 0.847) in the training cohort. CE-T1WI showed the greatest significance for staging NPC. Conclusion: Radiomics models can effectively distinguish early-stage from advanced-stage NPC patients, and Model A (CE-T1WI + T1WI + T2WI) showed the best classification performance.

## 1. Introduction

Nasopharyngeal carcinoma (NPC) is a common cancer of the head and neck with an endemic distribution, especially in southeastern China [1]. When its people emigrated to other countries, although the incidence of NPC decreased, it was also higher than that of the natives. Thus, although the pathogenesis remains unknown, it may be related to a combination of genetic, ethnic, and environmental factors [2].

Fortunately, NPC is highly sensitive to radiotherapy [3]. With the application of treatment paradigms involving intensity-modulated radiotherapy, the incidence of five years’ local recurrence and distant metastasis has significantly decreased [2,4,5]. Treatment options are closely related to the clinical stages of NPC [2,3]. Therefore, accurate clinical stages of NPC are crucial for treatment. At present, the staging of NPC mainly depends on the Union for International Cancer Control/American Joint Committee on Cancer (UICC/AJCC) tumor-node metastasis (TNM) staging system [2]. Magnetic resonance imaging (MRI) is widely used in diagnosing diseases in various body organs [6]. With better visibility than other existing imaging methods, it is considered the optimal approach for staging [7,8,9,10,11]. Clinical stages of NPC were assessed by radiologists based on MR images and clinical data according to the TNM staging system. However, for the patients at the same TNM stages, local recurrence and distant metastasis still occur in some of them under current staging methods, even when they are treated with the same strategies [12]. For these patients, current staging methods may not accomplish accurate clinical stages. This may be due to a loss of much information obtained in MR images through visual assessment, as well as the fact that differing experience of radiologists may influence staging accuracy. Thus, a new technique for accurately staging NPC is urgently needed.

With the development of biomedical imaging, MRI is also highly sophisticated, and there is no denying that MR images contain much information not visible for visual assessment [13]. By using high-throughput extraction of data-characterization algorithms, radiomics provides the opportunity to demonstrate the characteristics of tumors that are difficult for visual assessment and characterize intratumoral heterogeneity [14,15]. Artificial intelligence based on these characteristics has been employed to solve many medical problems, like biomedical image analysis and healthcare [13]. Therefore, many studies have investigated the potential of radiomics in predicting the preoperative stage, prognosis, response to treatment, and recurrence of tumors, with good performance found in lung cancer, breast cancer, and some abdominal cancers [16,17,18,19,20,21,22]. Radiomics has also achieved good performance in predicting distant metastasis, local recurrence, and progression-free survival (PFS) of NPC [23,24,25,26,27,28].

However, only the performance of radiomics to predict T stages or distinguish advanced clinical stages (stage III vs. IV) of NPC patients has been investigated; its ability to predict the clinical stages of NPC patients remains unknown [29,30]. Therefore, seven models were constructed to explore the capability of radiomics in staging NPC patients (clinical stage I–II vs. III–IVa) based on MR images.

## 2. Materials and Methods

### 2.1. Patients

Patients with pathologically confirmed NPC from January 2013 to December 2016 were enrolled in our study. The inclusion criteria were as follows: (1) primary NPC (stage I~IVa); (2) complete clinical data; (3) received MRI scans in our hospital within 2 weeks before treatment; (4) no history of chemotherapy or radiotherapy before the MRI scan; and (5) maximum lesion diameter larger than 10 mm. The exclusion criteria were as follows: (1) meanwhile combined with other cancers; (2) suffering from severe chronic wasting diseases; and (3) MR images with artifacts, faults, blurs, and disordered slices.

**Figure 1:**
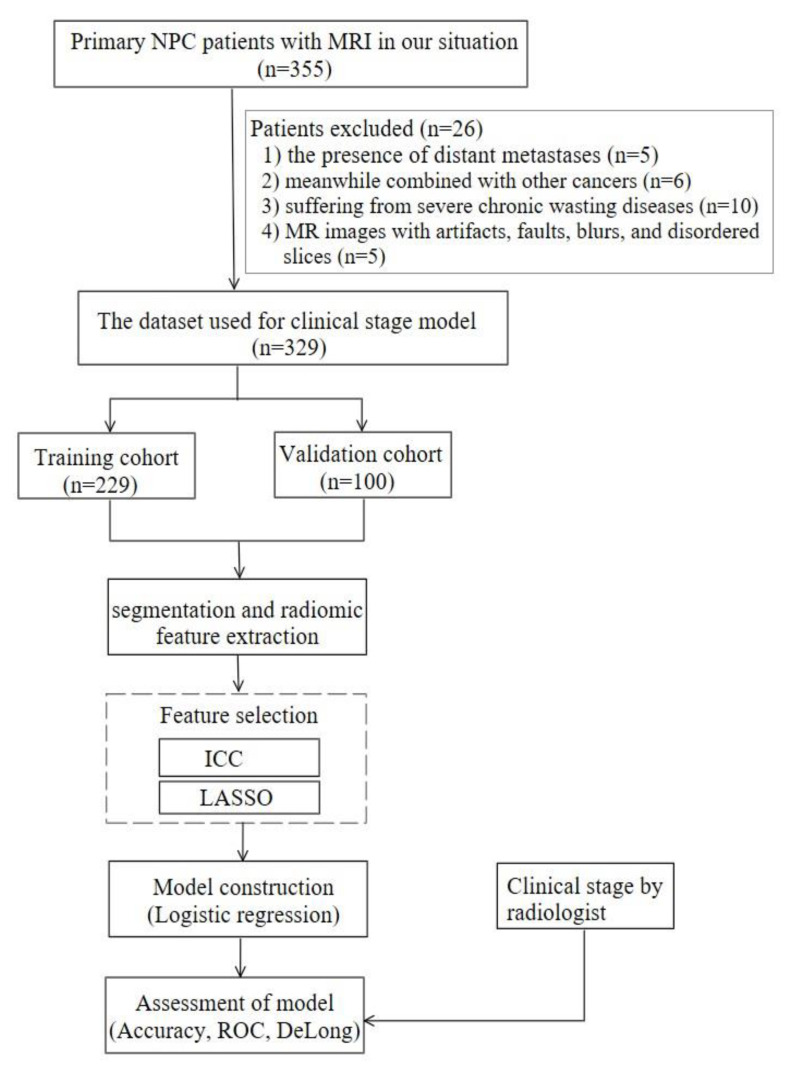
Flow chart of this study.

A total of 329 patients were recruited (mean age 49.80 ± 10.67 years, ranging from 15 to 76 years), made up of 234 males and 95 females. They were randomly divided into training and validation cohorts at a ratio of 7:3. Therefore, 229 patients were allocated to the training cohort and the other 100 were allocated to the validation cohort. Demographic and clinical data (age, gender, smoking, and drinking) were collected. The workflow of this study is presented in Figure 1.

### 2.2. Image Acquisition

All patients were scanned with the Siemens Magnetom Essenza 1.5-T MR scanner from the middle temporal lobe to the superior aperture of the thorax. Axial T1-weighted images (T1WI), T2-weighted images (T2WI), fast spin-echo T2-weighted images, and contrast-enhanced T1-weighted images (CE-T1WI) were performed on all patients. The MR imaging protocols were as follows: (1) axial T1WI (repetition time [TR]/echo time [TE] = 769/10 ms, number of excitation (NEX) = 1, and slice thickness = 5 mm); (2) axial T2WI (TR/TE = 6920/81 ms, NEX = 1, and slice thickness = 5 mm); (3) axial FSE T2WI (TR/TE = 4260/86 ms, NEX = 1, and slice thickness = 5 mm); and (4) axial CE-T1WI (TR/TE = 7.93/2.38 ms, NEX = 1, and slice thickness = 5 mm) were obtained after injecting 0.01 mmol/kg of gadopentetate dimeglumine through the median cubital vein at a speed of 2 mL/s. The slice thickness of all protocols was 5 mm.

### 2.3. Patient Restaging and Human Visual Assessment

All MR images and clinical data were separately reviewed by 2 experienced radiologists (with 20 years and 30 years of head and neck radiology experience, respectively). They re-evaluated the clinical stages into early-stage (I~II) and advanced-stage (III~IVa) according to the eighth edition of the UICC/AJCC TNM staging system [2]. Any differences were resolved through consensus.

In addition, another 2 radiologists (reader 1 and reader 2 with 5 years and 6 years of experience in head and neck MRI, respectively) were recruited to separately stage NPC based on MR images, and were all blinded to the patients’ clinical data. They then worked together to resolve differences by consensus.

### 2.4. Image Segmentation and Feature Extraction

MR images were all anonymously retrieved from the picture archiving and communication system (PACS). Image segmentation was performed by reader 1. The three-dimensional volume of interest that contained the whole primary tumor was obtained by stacking up the region of interest (ROI). This was manually delineated slice by slice around the outermost boundary of the tumor on axial sequences (T1WI, T2WI, and CE-T1WI separately) using 3D Slicer (version 4.10.2; http://www.slicer.org, accessed on 17 May 2019). To ensure the segmentation only contained tumor tissue, 3 mm inside the ROI was decreased with automated dilation and shrinkage.

Feature extraction was performed with the open-source Pyradiomics package (version 3.0.1; https://pyradiomics.readthedocs.io/en/latest/changes.html#pyradiomics-3-0-1, accessed on 3 June 2021). To standardize the voxel spacing, images were resampled to a voxel size of 1 × 1 × 1 mm^3^. After that, seven classes of radiomics features were extracted from the original images, including shape, first-order, gray-level co-occurrence matrix (GLCM), gray-level dependence matrix (GLDM), gray-level run length matrix (GLRLM), gray-level size zone matrix (GLSZM), and neighboring gray tone difference matrix (NGTDM) features. The same first-order and textural features were then extracted after applying wavelet (with 3 directions of wavelet decomposition: x, y, z) and Laplacian of Gaussian (LoG) (with sigma values of 0.5 mm, 1.0 mm, 1.5 mm, 2.0 mm) to the original images, respectively. Ultimately, 3669 features were extracted (1223 from each sequence). The detailed radiomics features are listed in Supplementary Materials File A.

### 2.5. Interobserver and Intraobserver Agreement

Forty patients (20 early-stage and 20 advanced-stage) were randomly chosen for repetitive tumor ROI segmentations, which were performed by reader 1 and reader 2 to explore interobserver stability. The same procedure was repeated by reader 1 in a 2-week period to evaluate intraobserver reproducibility. The intraclass correlation coefficient (ICC) was used to evaluate intraobserver and interobserver agreement, and ICC > 0.75 indicated satisfactory agreement. Therefore, only features with both intra- and interobserver ICC > 0.75 were chosen for further analysis and were standardized with z score normalization.

### 2.6. Dimensionality Reduction and Radiomics Feature Selection

To reduce potential overfitting of the radiomics features and avoid the curse of dimensionality when modeling, two steps were applied to select radiomics features in the training cohort. First, the independent samples *t* test or the Mann–Whitney U test was used to select potentially important features. Second, features with *p* < 0.05 from the first step were kept and input to the least absolute shrinkage and selection operator (LASSO) classifier, with penalty parameter tuning conducted by 10-fold cross-validation, and features with non-zero coefficients were selected to build radiomics signatures [31,32]. The radiomics score (Rad-score) for each patient was calculated using a linear combination of selected features that were weighted by their respective LASSO coefficients.

### 2.7. Construction of the Radiomics Model

Logistic regression, a classical machine learning method, was used to construct seven radiomics models (named A, B, C, D, E, F, and G) for staging NPC patients. Models A, B, C, and D were built with radiomics signatures selected from combined sequences (CE-T1WI + T1WI + T2WI, CE-T1WI + T1WI, T1WI + T2WI, and CE-T1WI + T2WI, respectively). Models E, F, and G were built with radiomics signatures selected from single sequences (CE-T1WI, T1WI, and T2WI, respectively).

**Figure 2:**
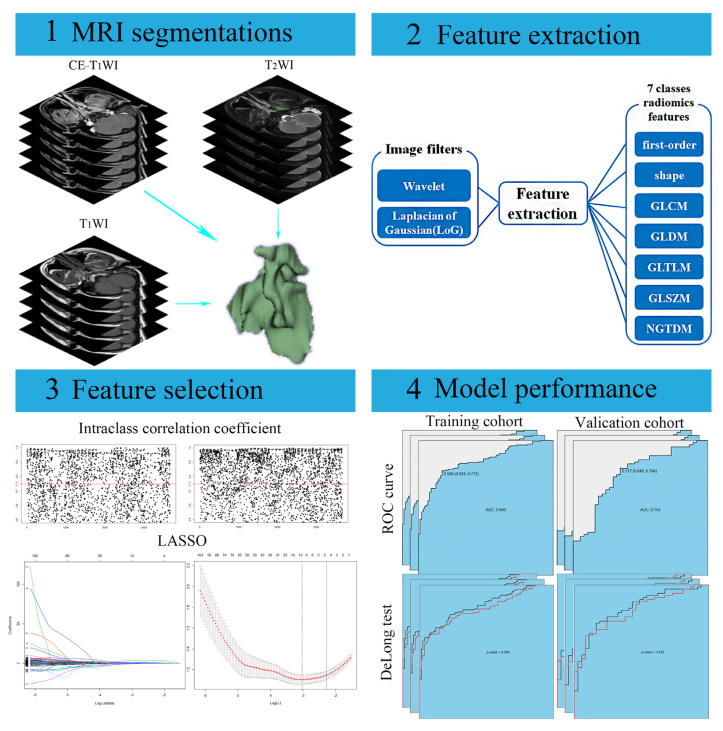
The workflow of model construction and evaluation. Note: MRI, Magnetic resonance image. CE-T1WI, Contrast-enhanced T1-weighted image. T1WI, T1-weighted image. T2WI, T2-weighted image. GLCM, Gray-level co-occurrence matrix. GLDM, Gray-level dependence matrix. GLRLM, Gray-level run length matrix. GLSZM, Gray-level size zone matrix. LoG, Laplacian of Gaussian. NGTDM, Neighboring gray tone difference matrix. LASSO, Least absolute shrinkage and selection operator. ROC, Receiver operator characteristic.

Accuracy, sensitivity, specificity, and the area under the receiver operating characteristic curve (AUC) values were used to evaluate models’ performance. ROC curves were drawn to display and compare the performance of different models. The DeLong test was used to analyze significant differences between models. The workflow of model construction and evaluation is shown in Figure 2.

### 2.8. Statistics Analysis

All statistical analyses of radiomics features were performed by R software (Version 3.6.0, https://www.r-project.org/, accessed date 29 March 2019). The following R packages were utilized: psych package (version 2.1.9) for the calculation of ICCs, glmnet package (version 4.1-1) for LASSO, pROC package (version 1.17.0.1) for ROC curves, and e1071 package (version 1.7-6) for the DeLong test.

The statistical analysis of demographic features was performed by IBM SPSS software (version 21.0). Continuous variables were compared using Mann–Whitney U tests and categorical variables were compared using chi-square tests. For all tests, a two-sided *p* value less than 0.05 was considered statistically significant.

## 3. Results

### 3.1. Patient Characteristics

Patients’ demographic information is recorded in Table 1. No significant differences were observed between the training and validation cohorts in terms of age, gender, smoking, drinking, T stage, N stage, or clinical stage.

### 3.2. Interobserver and Intraobserver Agreement

Only 407 features from CE-T1WI, 390 features from T1WI, and 338 features from T2WI, whose ICC scores were all greater than 0.75, were selected. In total, 1135 radiomics features were selected for the following analysis, listed in Supplementary Materials File B.

### 3.3. Dimensionality Reduction and Radiomics Feature Selection

All 1135 radiomics features showed significant differences (*p* < 0.05) when tested by independent samples *t* tests or Mann–Whitney U tests and were used for LASSO regression. This was followed by the selection of 13, 9, 7, and 9 features derived from combined sequences (CE-T1WI + T1WI + T2WI, CE-T1WI + T1WI, T1WI + T2WI, and CE-T1WI + T2WI) to construct Models A, B, C, and D respectively. Ten, seven, and six features derived from single sequences (CE-T1WI, T1WI, and T2WI) were selected to construct Models E, F, and G respectively. The selected features for each model and calculation formulas for Rad-scores are shown in Table 2.

### 3.4. Performance of Different Models and Radiologists

**Figure 3:**
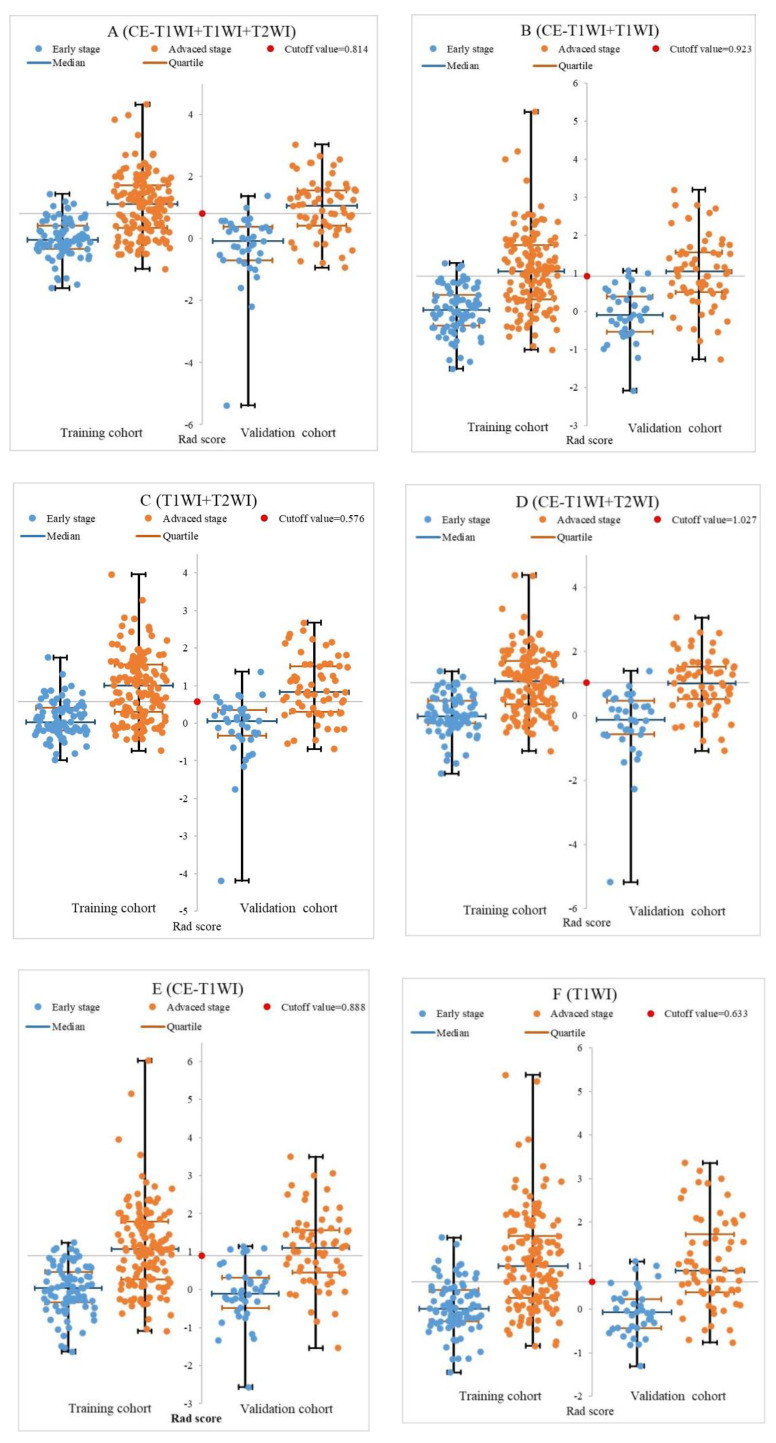

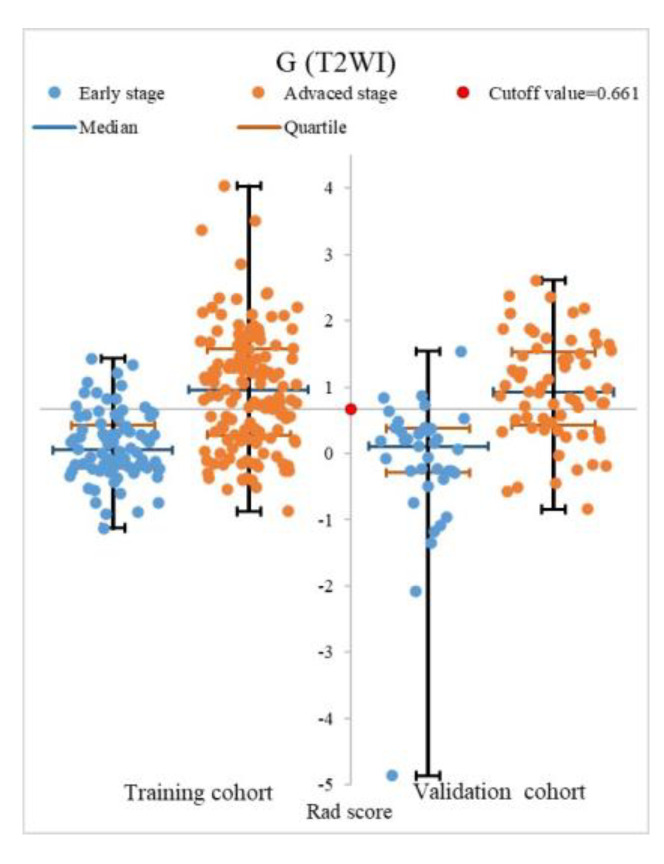
The Rad-scores for each patient in the training and validation cohorts regarding the classification of stage I–II and stage III–IVa NPC. Note: In all radiomics models, the median Rad-scores in the advanced stage were all higher than those in the early stage.

Median values and interquartile ranges of the Rad-scores in the training and validation cohorts are listed in Table 3. All showed potential abilities in differentiating stage I–II from stage III–IVa both in training (all *p* < 0.001) and validation (all *p* < 0.001) cohorts, with the Rad-scores of the latter being much higher. The Rad-scores of seven models for each patient in the training and validation cohorts regarding the classification of stage I–II and stage III–IVa NPC are depicted in Figure 3.

**Figure 4:**
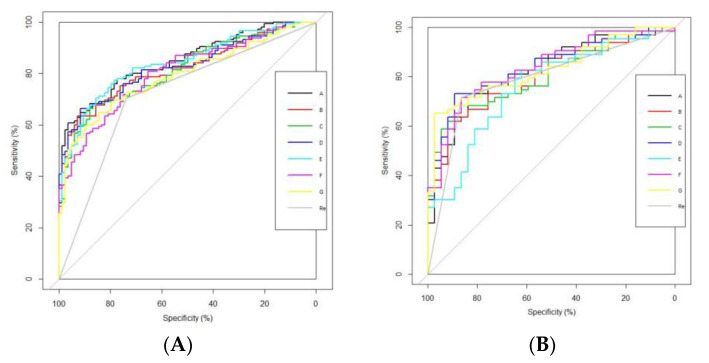
ROC curves of different models and readers. Note: (**A**) shows the ROC curves of different radiomics models and readers in the training cohort; Model A showed the highest AUC value. (**B**) shows the ROC curves of different radiomics models and readers in the validation cohort. A, CE-T1WI + T1WI + T2WI. B, CE-T1WI + T1WI. C, T2WI + T1WI. D, CE-T1WI + T2WI. E, CE-T1WI. F, T1WI. G, T2WI. Re, readers.

The classification performance of readers and seven radiomics models are listed in Table 4. The ROC curves of different radiomics models and readers are shown in Figure 4. When comparing the performance of human visual assessment with all radiomics models using the DeLong test, readers (with an AUC, accuracy, specificity, and sensitivity of 0.721, 0.716, 0.738, and 0.703, respectively) showed the worst performance. When comparing the performance of Model A with that of the other radiomics models using the DeLong test, Model A (with an AUC, accuracy, specificity, and sensitivity of 0.847, 0.729, 0.571, and 0.820, respectively) showed the best performance in the training cohort. However, there were no significant differences for any of the validation cohorts. Furthermore, there were no significant differences when comparing the performance of other models in pairs.

In the models constructed with features derived from combined sequences, although Model A showed the best classification performance, there were no significant differences between Models A and D (CE-T1WI + T2WI, the AUC, accuracy, specificity, and sensitivity in the training cohort were 0.826, 0.751, 0.679, and 0.793, respectively) in the training cohorts. Model C (T1WI + T2WI) showed the worst classification performance, with the lowest AUC value of 0.812 in the training cohort (the accuracy, specificity, and sensitivity were 0.703, 0.595, and 0.766, respectively).

In the models constructed with features derived from a single sequence, Model E (CE-T1WI) showed the best classification performance in the training cohort (the AUC, accuracy, specificity, and sensitivity were 0.839, 0.764, 0.667, and 0.821, respectively), which ranked second to that of Model A. However, there were no significant differences between Models A and E in either the training cohort or the validation cohort. Model G (T2WI) showed the worst classification performance in the training cohort (with an AUC, accuracy, specificity, and sensitivity of 0.791, 0.716, 0.631, and 0.766, respectively).

## 4. Discussion

In this retrospective study, the median Rad-scores in advanced-stage NPC were all higher than those in early-stage NPC, which indicated great potential of radiomics features in differentiating early-stage NPC from advanced-stage NPC. However, the reason was unknown, maybe due to the fact that every model’s features consisted of two or more features related to the shape of lesions, and most of the advanced-stage tumor lesions were bigger than that of early-stage. Thus, median Rad-scores in advanced-stage NPC were all higher than those in early-stage NPC.

As expected, all radiomics models showed better classification performance than that of visual assessment by a set of radiologists with less experience, with Model A (CE-T1WI + T1WI + T2WI) the best (AUC: 0.847 in the training cohort and 0.824 in the validation cohort). This may be due to the complicated anatomy of the head and neck, undefined involvement of surrounding tissues, and ambiguous metastasis of lymph nodes, which make it difficult for young radiologists to accurately identify clinical stages. However, there were no significant differences in validation cohorts when comparing classification performance of visual assessment with that of radiomics models. As we all know, adjacent invasion tissues and metastatic neck LNs were highly relevant to TNM stage of NPC patients, thus MR images of invasion tissue and metastatic neck LNs contained much information related to clinical stages. However, in our study, ROIs did not cover the adjacent invasion tissues and metastatic neck LNs. The ignorance of these images may have resulted in no significant differences in validation cohorts when comparing classification performance of radiomics models with that of visual assessment. However, given that most AUC values for radiomics models were higher than that of visual assessment, it cannot be denied that radiomics models showed better classification performance.

By extracting quantitative parameters from MR images, machine learning classifiers can minimize the influence of radiologists’ differing experience and accurately characterize intratumoral heterogeneity [13]. However, for the validation cohort, no significant differences were observed between any of the models, which may be due to its small sample size and the imbalanced sample size for each stage of NPC patients (the number of advanced-stage patients was almost twice that of early-stage). As shown in Table 2, there were many identical features selected for each model, especially for models A, B, D and E. This was considered the reason for no significant differences between the validation cohorts of each model and training cohorts of model A, D, and E. Features related to the shape of tumors were selected in all models, the reason being that most tumor lesions in advanced-stage were bigger than that in early-stage, and were considered the most significant features for clinical staging.

Furthermore, CE-T1WI was considered the most significant sequence for staging NPC. As we can see, Model E (CE-T1WI) showed the best classification performance in the models constructed from a single sequence, which ranked second only to Model A (with no significant differences between them). This may be due to the concentration of contrast material in CE-T1WI, which revealed the blood supply of the tumor, so CE-T1WI can provide more information for clinical staging of NPC. However, features derived from T1WI and T2WI should not be ignored, since they helped Model A to achieve the best classification performance. We thought this was due to different sequences of MR images reflecting different characteristics of the tumor tissue, meaning that radiomics can extract totally different features from different sequences, which played an important role in accurately identifying stages [6].

One study showed similar conclusions to ours; its CE-T1WI showed better classification performance than that of T2WI, and signatures from CE-T1WI + T2WI showed the best performance (AUC: 0.850 in training cohorts and 0.849 in validation cohorts) [33]. However, in our study, the classification performance of CE-T1WI+T2WI showed no significant difference from that of CE-T1WI, which may be owing to the different subjects and smaller sample size (127 head and neck squamous cell carcinoma patients) in that study.

To our knowledge, our study is the first to differentiate early-stage NPC patients from advanced-stage NPC patients. A previous study established a weakly supervised deep learning network, with 1138 cases of images (T1WI, T2WI, and CE-T1WI) inputted to train this model which achieved good performance in automated T staging of NPC (the average AUC value of different T stages was 0.943) and showed no significant differences in PFS and overall survival with those of the TNM stage system [30]. Although deep learning showed great potential in automatic T staging, an accurate clinical stage of NPC was still not achieved due to the unknown N stage in that study. Moreover, large numbers of MR images are usually needed to train deep learning models, which makes it difficult to be replicated and verified.

Another previous study established five Convolutional Neural Network models combined with transfer learning to differentiate advanced stages (III and IV) of NPC. Patches of CE-T1WI and T2WI images containing tumors, metastatic lymph nodes, and their adjacent tissues were inputted for training. The predicted stages were finally obtained by software voting, with the combined model showing better classification performance (accuracy: 0.81) than that of the TNM stage system and the traditional radiomics model under the same experimental conditions [29]. However, approximately only 200 patients were enrolled in that study, the accuracy of the model may have been compromised, and early-stage patients were not included. In addition, deep learning is still considered a black-box technique and should be interpretable for crucial application. In our study, features were directly extracted from primary tumors to predict the overall clinical stage, which decreased the influences of different radiologists and unknown N stage in accurate staging. The model was a classical machine learning method with specific algorithms and achieved good performance (AUC: 0.847) in differentiating early-stage NPC from advanced-stage NPC, which highlighted the great potential of radiomics in predicting the clinical stage of NPC.

Although good performance was achieved in our study, there were still inaccurate staging cases. The reasons for these may be as follows: (1) Sample sizes were imbalanced for the two groups in this study. (2) The involvement of the parapharyngeal space was proven to be related to T stage and prognosis of NPC [34], thus only tumor tissues were obtained; removing the related tissues and metastatic lymph nodes may decrease accuracy. (3) This study directly predicted clinical stages instead of T and N stages, which may ignore the specific sites of invasion and lead to inaccuracy. (4) Images in the coronal plane and sagittal plane were not included in the model construction, which may lead to the loss of some imaging information.

There are some limitations in this study: (1) There was no external cohort to verify these radiomics models. (2) Only a two-stage classification framework was performed; the effect of radiomics in differentiating more detailed clinical stages was lacking. (3) A slice thickness of 5 mm may miss the minor invasion and lead to inaccurate stages. (4) Only patients admitted from 2013–2016 were enrolled in this study, because it was the first part of our research; a 5-year follow-up was needed for the rest. We plan to enlarge and balance the sample sizes of each stage to further investigate the ability of radiomics to predict the prognosis and differentiate more detailed clinical stages of NPC.

## 5. Conclusions

In conclusion, radiomics models showed great potential in distinguishing early-stage (I–II) from advanced-stage (III–IVa) NPC patients, and Model A (CE-T1WI + T1WI + T2WI) performed the best. Furthermore, CE-T1WI showed the highest significance in staging NPC. However, imbalanced sample sizes, ignorance of adjacent tissue and LNs, and a single machine learning classifier may all lead to inaccurate staging cases, and also resulted in no significant differences for validation cohorts between models and visual assessment. Thus, we plan to enlarge and balance our sample size, extend the ROI to contain adjacent tissue and LNs, and compare the classification performance of different machine learning classifiers and deep learning, to find an accurate staging method for NPC.

## Figures and Tables

**Table 1:** Clinical characteristics of the training cohort and validation cohort.

	Training Cohort	Validation Cohort	*p*
	n = 229	n = 100	
Age(years)	50.341 ± 10.274	48.570 ± 11.488	0.241
Gender			0.817
Male	162 (70.742%)	72 (72.000%)	
Female	67 (29.258%)	28 (28.000%)	
Smoking			0.367
Yes	120 (52.402%)	47 (47.000%)	
No	109 (47.598%)	53 (53.000%)	
Drinking			0.186
Yes	105 (45.852%)	38 (38.000%)	
No	124 (54.148%)	62 (62.000%)	
T stage			0.528
T1	36 (15.721%)	17 (17.000%)	
T2	90 (39.301%)	42 (42.000%)	
T3	63 (27.511%)	20 (20.000%)	
T4	40 (17.467%)	21 (21.000%)	
N stage			0.624
N0	49 (21.397%)	24 (24.000%)	
N1	99 (43.231%)	44 (44.000%)	
N2	55 (24.017%)	18 (18.000%)	
N3	26 (11.354%)	14 (14.000%)	
Clinical stage			0.701
I	13 (5.677%)	6 (6.000%)	
II	71 (31.004%)	31 (31.000%)	
III	82 (35.808%)	30 (30.000%)	
IV	63 (27.511%)	33 (33.000%)	

Note: Continuous variables were compared using Mann–Whitney U tests and categorical variables were compared using chi-square tests. *p* < 0.05 indicated significant differences.

**Table 2:** The selected features for each model and calculation formulas for radiomics scores.

Sequence	Numbers of Selected Features	Selected Features	Coefficients
A (CE-T1WI + T1WI + T2WI)	13	Intercept	−2.29674179
		CE-T_1_WI_Shape_LeastAxisLength	0.74147306
		CE-T_1_WI_Shape_Maximum2DDiameterSlice	0.85277531
		CE-T_1_WI_LoG.sigma.2.0.mm.3D_GLSZM_ZoneEntropy	3.20584709
		T_1_WI_Wavelet.HLH_GLCM_InverseVariance	0.13071358
		T_2_WI_Wavelet.LLL_firstorder_10Percentile	−0.10023274
		CE-T_1_WI_Wavelet.HLL_firstorder_Mean	0.01520543
		CE-T_1_WI_Wavelet.HHL_GLCM_Imc1	0.43246473
		CE-T_1_WI_Wavelet.LLL_GLCM_ClusterShade	0.01804038
		CE-T_1_WI_NGTDM_Busyness	0.08374613
		T_1_WI_LoG.sigma.0.5.mm.3D_GLSZM_GrayLevelNonUniformity	0.20730698
		T_1_WI_Wavelet.LLH_GLCM_MaximumProbability	−0.57803318
		T_2_WI_LoG.sigma.2.0.mm.3D_firstorder_Median	0.30651800
		T_2_WI_Wavelet.LLL_firstorder_Median	−0.81238312
B (CE-T1 WI + T1WI)	9	Intercept	−3.55286386
		CE-T_1_WI_Shape_LeastAxisLength	1.19584018
		CE-T_1_WI_Shape_Maximum2DDiameterSlice	0.63791229
		CE-T_1_WI_LoG.sigma.2.0.mm.3D_GLSZM_ZoneEntropy	3.25384123
		CE-T_1_WI_Wavelet.HLL_firstorder_Mean	0.08296501
		CE-T_1_WI_Wavelet.HHL_GLCM_Imc1	0.30143138
		CE-T_1_WI_Wavelet.LLL_GLCM_ClusterShade	0.02020103
		CE-T_1_WI_NGTDM_Busyness	0.12360246
		T_1_WI_LoG.sigma.0.5.mm.3D_GLSZM_GrayLevelNonUniformity	0.23228234
		T_1_WI_Wavelet.LLH_GLCM_MaximumProbability	−0.67821075
C (T2WI + T1WI)	7	Intercept	−0.45953586
		T_2_WI_LoG.sigma.2.0.mm.3D_firstorder_Median	0.66448895
		T_2_WI_Wavelet.LLL_firstorder_Median	−0.11340379
		T_1_WI_Shape_LeastAxisLength	0.07440248
		T_1_WI_Shape_Maximum2DDiameterSlice	1.30038478
		T_1_WI_Shape_MinorAxisLength	0.04818318
		T_1_WI_LoG.sigma.0.5.mm.3D_GLSZM_GrayLevelNonUniformity	0.48611908
		T_1_WI_Wavelet.LLH_GLCM_MaximumProbability	−0.03052694
D (CE-T1 WI + T2WI)	9	Intercept	−3.66635179
		CE-T_1_WI_Shape_LeastAxisLength	0.64460654
		CE-T_1_WI_Shape_Maximum2DDiameterSlice	0.96018848
		CE-T_1_WI_LoG.sigma.2.0.mm.3D_GLSZM_ZoneEntropy	3.95808134
		T_2_WI_Wavelet.LLL_firstorder_10Percentile	−0.21538833
		CE-T_1_WI_Wavelet.HHL_GLCM_Imc1	0.30330139
		CE-T_1_WI_Wavelet.LLL_GLCM_ClusterShade	0.00170924
		CE-T_1_WI_NGTDM_Busyness	0.14266115
		T_2_WI_LoG.sigma.2.0.mm.3D_firstorder_Median	0.36736992
		T_2_WI_Wavelet.LLL_firstorder_Median	−0.49868865
E (CE-T1WI)	10	Intercept	−4.66905571
		Shape_LeastAxisLength	1.33242867
		Shape_Maximum2DDiameterSlice	0.84780188
		LoG.sigma.2.0.mm.3D_GLSZM_ZoneEntropy	3.51313223
		Wavelet.LHL_GLCM_InverseVariance	−0.16837098
		LoG.sigma.2.0.mm.3D_GLCM_InverseVariance	−0.03184454
		Wavelet.HLL_firstorder_Mean	0.08653895
		Wavelet.LHL_GLDM_DependenceNonUniformityNormalized	0.27658583
		Wavelet.HHL_GLCM_Imc1	0.42044564
		Wavelet.LLL_GLCM_ClusterShade	0.06160728
		NGTDM_Busyness	0.29172003
F (T1WI)	7	Intercept	−2.53872918
		Shape_LeastAxisLength	1.30506559
		Shape_Maximum2DDiameterSlice	1.07343910
		Wavelet.HLH_GLCM_InverseVariance	1.31055892
		LoG.sigma.0.5.mm.3D_GLSZM_GrayLevelNonUniformity	0.55947887
		Wavelet.LLH_GLCM_MaximumProbability	−0.76719457
		Wavelet.HLH_GLCM_Imc1	0.07350668
		Wavelet.HHL_GLSZM_GrayLevelNonUniformity	0.06013319
G (T2WI)	6	Intercept	−0.70475300
		Shape_LeastAxisLength	0.32835260
		Shape_Maximum2DDiameterSlice	1.44163325
		Shape_MinorAxisLength	0.34220331
		LoG.sigma.2.0.mm.3D_firstorder_Median	0.79732259
		Wavelet.LHL_GLSZM_GrayLevelNonUniformity	0.04805125
		Wavelet.LLL_firstorder_Median	−0.08102625

Note: Eight different combinations of low-pass (L) and high-pass (H) filters wavelet transformations were used (i.e. LLH, LHL, LHH, HLL, HLH, HHL, HHH, LLL). GLCM, Gray-level co-occurrence matrix. GLDM, Gray-level dependence matrix. GLRLM, Gray-level run length matrix. GLSZM, Gray-level size zone matrix. LoG, Laplacian of Gaussian. NGTDM, Neighboring gray tone difference matrix.

**Table 3:** The median of Rad-scores based on different sequences.

	Training Cohort			Validation Cohort			
	Stage I–II	Stage III–IV	*p*		Stage I–II	Stage III–IV	*p*
A (CE-T1WI + T1WI + T2WI)	−0.044 (−0.334–0.414)	1.122 (0.342–1.719)	<0.001	−0.080 (−0.701–0.376)		1.052 (0.421–1.555)	<0.001
B (CE-T1WI + T1WI)	0.044 (−0.371–0.438)	1.059 (0.319–1.742)	<0.001	−0.089 (−0.541–0.390)		1.046 (0.505–1.559)	<0.001
C (T1WI + T2WI)	0.027 (−0.200–0.413)	1.006 (0.305–1.555)	<0.001	0.059 (−0.335–0.351)		0.824 (0.305–1.512)	<0.001
D (CE-T1WI + T2WI)	−0.019 (−0.245–0.463)	1.072 (0.352–1.697)	<0.001	−0.125 (−0.582–0.463)		1.011 (0.514–1.518)	<0.001
E (CE-T1WI)	0.034 (−0.336–0.471)	1.062 (0.267–1.791)	<0.001	−0.108 (−0.483–0.315)		1.100 (0.454–1.564)	<0.001
F (T1WI)	0.012 (−0.277–0.442)	0.990 (0.256–1.682)	<0.001	−0.066 (−0.429–0.233)		0.882 (0.384–1.720)	<0.001
G (T2WI)	0.047 (−0.218–0.420)	0.962 (0.276–1.577)	<0.001	0.106 (−0.293–0.375)		0.924 (0.428–1.529)	<0.001

Note: Data are expressed as the median (interquartile range); *p* < 0.05 indicates significant differences.

**Table 4:** The performance of 7 radiomics models in staging NPC.

		95%CI	AUC	Specificity	Sensitivity	Accuracy	PPV	NPV	Z1	*P*1	Z2	*P*2
Reader	training	―	0.721	0.738	0.703	0.716	0.823	0.590	―	―	―	―
	validation	―	0.790	0.865	0.714	0.770	0.900	0.640	―	―	―	―
A	training	[0.799–0.895]	0.847	0.571	0.820	0.729	0.768	0.649	3.725	0.000 *	―	―
	validation	[0.741–0.906]	0.824	0.676	0.794	0.750	0.806	0.658	0.704	0.481	―	―
B	training	[0.777–0.879]	0.820	0.560	0.814	0.721	0.761	0.635	2.775	0.006 *	1.992	0.046 *
	validation	[0.757–0.914]	0.803	0.568	0.810	0.720	0.761	0.636	0.258	0.797	0.796	0.426
C	training	[0.768–0.873]	0.812	0.595	0.766	0.703	0.766	0.595	2.610	0.009 *	2.560	0.010 *
	validation	[0.718–0.887]	0.804	0.514	0.809	0.700	0.739	0.613	0.308	0.758	0.658	0.511
D	training	[0.774–0.878]	0.826	0.679	0.793	0.751	0.810	0.655	3.050	0.002 *	1.814	0.070
	validation	[0.757–0.914]	0.836	0.703	0.762	0.740	0.814	0.634	0.953	0.341	−0.674	0.500
E	training	[0.790–0.891]	0.839	0.667	0.821	0.764	0.810	0.683	3.271	0.001 *	0.433	0.665
	validation	[0.656–0.853]	0.760	0.622	0.794	0.730	0.781	0.639	0.953	0.341	1.523	0.128
F	training	[0.747–0.858]	0.803	0.583	0.841	0.747	0.777	0.681	2.299	0.022 *	2.644	0.008 *
	validation	[0.759–0.915]	0.837	0.568	0.825	0.730	0.765	0.656	0.963	0.336	−0.533	0.594
G	training	[0.734–0.848]	0.791	0.631	0.766	0.716	0.782	0.609	2.015	0.044 *	3.363	0.001 *
	validation	[0.749–0.907]	0.828	0.595	0.794	0.720	0.769	0.629	0.799	0.425	−0.138	0.891

Note: Z1 and *P*1, the performance of readers compared with that of radiomics models using the DeLong test. Z2 and *P*2, the performance of Model A compared with that of the other radiomics models using the DeLong test. * *p* < 0.05. AUC, area under the curve. CI, confidence interval. PPV, positive predictive value. NPV, negative predictive value. A, CE-T1WI + T1WI + T2WI. B, CE-T1WI + T1WI. C, T2WI + T1WI. D, CE-T1WI + T2WI. E, CE-T1WI. F, T1WI. G, T2WI.

## Data Availability

The datasets used and/or analyzed during the current study are available from the corresponding author upon reasonable request.

## Supplementary Materials

The following supporting information can be downloaded at: https://www.mdpi.com/article/10.3390/diagnostics13020300/s1, Supplement A: All detailed original radiomics features. Supplement B: All radiomics features with ICC > 0.75 for both intra- and interobserver agreement.
